# Special Interest- what are trainees doing in the West Midlands?

**DOI:** 10.1192/bjo.2021.512

**Published:** 2021-06-18

**Authors:** Nidhi Gupta, Fiona Hynes

**Affiliations:** Birmingham and Solihull Mental Health NHS Foundation Trust

## Abstract

**Aims:**

The aim of this survey was to find out how Speciality trainees used their special interest sessions, using multiple choice and open questions

**Background:**

The ST (Speciality Training) curriculum recognises that it is desirable that all higher trainees gain additional experiences that may not be available in their clinical placement. Two sessions every week must be devoted during each year of Speciality training for such personal development, which includes research or to pursue special interests. Special interest sessions are defined as “a clinical or clinically related area of service which cannot be provided within the training post but which is of direct relevance to the prospective career pathway of the trainee”. This experience must be appropriately managed, supervised and assessed.

**Method:**

We conducted a survey of Speciality trainees in the West Midlands region across all psychiatric specialities using an online survey. The survey was open for one month period in January 2021 and reminders were sent intermittently. Following survey closure, quantitative data were analysed using Google Forms and Excel. Qualitative data were collated and reviewed to identify relevant themes.

**Result:**

47 of the total 82 Speciality trainees in all psychiatric specialities including dual trainees responded. Maximum response rate was from General adult/Dual trainees who form the bulk of Speciality trainees. Most trainees discussed their special interest with their supervisors and included this in learning plans. 79% were able to have a weekly session. Most sessions were devoted to gaining additional clinical experience, medical education, gaining leadership competencies and completion of further post graduate qualifications. The majority of trainees chose special interest sessions in their own trust, however 45% had difficulty getting released from their clinical commitments. Trainees demonstrated evidence in their portfolio by reflection, WPBA and reflective notes. Trainees were positive about their experiences and requested more support to access sessions locally.

**Conclusion:**

The Future Doctor report (HEE 2020) recognised that our Future Doctors must have a broad range of generalist skills to meet the population needs, therefore it is essential that doctors in training are supported by trainers and trusts to access special interest sessions to ensure that they achieve a broad range of competencies. To signpost trainees we have developed a booklet advertising available opportunities for ST trainees and other services may wish to consider this.

